# Evolutionary behaviour of bacterial prion-like proteins

**DOI:** 10.1371/journal.pone.0213030

**Published:** 2019-03-05

**Authors:** Paul M. Harrison

**Affiliations:** Department of Biology, McGill University, Montreal, QC, Canada; University of Lausanne, SWITZERLAND

## Abstract

Prions in eukaryotes have been linked to diseases, evolutionary capacitance, large-scale genetic control and long-term memory formation. In bacteria, constructed prion-forming proteins have been described, such as the prion-forming protein recently described for *Clostridium botulinum* transcription terminator *Rho*. Here, I analyzed the evolution of the *Rho* prion-forming domain across bacteria, and discovered that its conservation is sporadic both in the *Clostridium* genus and in bacteria generally. Nonetheless, it has an apparent evolutionary reach into eight or more different bacterial phyla. Motivated by these results, I investigated whether this pattern of wide-ranging evolutionary sporadicity is typical of bacterial prion-like domains. A measure of coverage of a domain (**C**) within its evolutionary range was derived, which is effectively a weighted fraction of the number of species in which the domain is found. I observe that occurrence across multiple phyla is not uncommon for bacterial prion-like protein domain families, but that they tend to sample of a low fraction of species within their evolutionary range, like *Rho*. The *Rho* prion-like domain family is one of the top three most widely distributed prion-like protein domain families in terms of number of phyla. There are >60 prion-like protein domain families that have at least the evolutionary coverage of *Rho*, and are found in multiple phyla. The implications of these findings for evolution and for experimental investigations into prion-forming proteins are discussed.

## Background

Prions were originally identified as proteinaceous infectious particles made from the prion protein PrP-Sc that causes devastating neurological diseases in mammals. Prions are particles that propagate alternative states of proteins, through co-option of further copies of the same proteins. In the yeast *Saccharomyces cerevisiae*, these alternative states can be transmitted sustainably during budding, mating and artificial protocols in the laboratory. Yeast prions have been linked to disease-like states, evolutionary capacitance, and large-scale genetic control. The first well-characterized yeast prions, that underlie the [PSI+] and [URE3] prion states, are propagating amyloids (*i*.*e*., fibrillar beta-sheet aggregates) of the proteins Sup35p and Ure2p. The protein Sup35p functions as part of the translation termination complex. Formation of [PSI+] prions reduces the efficiency of translation termination and increases levels of nonsense-codon read-through [1, 2]. Such read-through has been shown to be a potential mechanism to uncover cryptic genetic variation [3, 4]. [URE3] causes upregulation of poor nitrogen source usage, even when rich sources are available [5–7]. Prion variants may be considered as diseases of *S*. *cerevisiae* in some contexts [8, 9]. The [MOT3+] prion, has been shown to have a possible function in controlling transition to multicellularity in *S*. *cerevisiae* [10]. There are now >10 known prions of *S*. *cerevisiae* that are propagated by amyloids [11, 12]. Prion-forming proteins have also been observed in the fungi *Podospora anserina* and fission yeast [13, 14]. Common compositional features of almost all amyloid-based budding yeast prions is bias for asparagine (N) and/or glutamine (Q) residues, and a high degree of intrinsic disorder [15]. Glutamine and asparagine seem to have different influences on prion formation: Ns promote benign prion formation, whereas excess Q composition can lead to formation of toxic non-amyloid conformers [16]. Several algorithms have been developed for annotating regions in proteins with high potential prion propensity [17–20].

The original PrP domain in mammals is not biased in this way, and is deeply conserved since a PrP founder gene likely emerged in chordates [21–23]. The [PSI+] prion has an N/Q bias that is conserved across fungal clades that diverged >1 billion years ago, with only eight other proteins showing such phylogenetically deep conservation of yeast-prion-like character [24]. A large population of yeast-prion-like proteins emerged early in the evolution of the budding yeasts *Saccharomycetes*, as a result of mutational trends that led to the formation of more polyasparagine runs, thus providing an evolutionary ‘test set’ out of which several prion-forming domains seem to have arisen [25]. A large fraction of known yeast prion-forming proteins (>40%) were predicted to maintain their prion-like status in more than half of the species in the *Saccharomycetes* clade [25]. It is not uncommon for eukaryotic proteomes to bear large numbers of these domains; in many fungal species, these simple, repetitive domains appear to arise because of general mutational biases for formation of N-rich and Q-rich sequences, rather than precise functional roles linked to their prion-like nature *per se* [25]. The slime mold *Dictyostelium* has greater than one fifth of its proteins containing PLDs or algorithmic prion predictions [26, 27] and there is evidence it has evolved a mechanism to subvert aggregate/prion formation [27, 28]. Other organisms that have high levels of prion-like proteins in them include *Drosophila melanogaster*, *Plasmodium falciparum* and the leech *Helobdella robusta* [26, 29]. Several other, yeast-prion-like proteins have been linked to neurodegenerative pathomechanisms in humans [30–32] or to long-term memory formation in *Aplysia* and fruit flies [33, 34]. Predicted prions have been detected across all the domains of life [19], including thousands in viruses and phages [35, 36].

Evidence for intra-cellular prion-like amyloids in bacteria has also been accumulating. RepA-WH1, a construct of the RepA protein that is itself able to form amyloids causes an artificial amyloid proteinopathy when expressed in *Escherichia coli* [37]. Bacteria have also been demonstrated to be able to propagate a yeast prion [38]. The bacteriotoxin microcin E492 has an amyloid prion-like alternative form whose propagation can be induced *in vivo* by an *in vitro* synthetic aggregate, or by exogenous addition of culture medium containing the amyloid form [39]. A survey of over 800 bacterial proteomes using a simple prion prediction algorithm discovered >2000 potential bacterial prions linked to diverse functional roles such as cell adaptability and invasion [40, 41].

Yuan & Hochschild [42] reported the construction of prion propagation for bacterial sequences expressed in bacterial cells, using a domain of transcription termination factor *Rho* from *Clostridium botulinum*. Rho functions in transcription termination; it binds to the transcription terminator pause site and is essential for transcription in prokaryotes. Experiments demonstrated amyloid formation for both full-length *Clostridium botulinum Rho* (*Rho*-bot), and for truncations or constructs, performed using heterologous expression in the Gram-negative species, *Escherichia coli*, and in the eukaryote budding yeast *S*. *cerevisiae*. Prion propagation was studied using hybrid constructs of the *Rho*-bot prion-like domain attached to the C-terminal transcription termination factor part of *E*. *coli Rho* protein. Genome-wide changes in the transcriptome were caused by prion formation and propagation arising from this chimeric protein. In this paper, they also pointed out that the prion-like domain of *Rho* appears in several diverse bacteria.

Here, I have performed a detailed analysis of the evolution of the prion(-like) domain of *Rho*. I discover that this prion domain is sporadically conserved in its own *Clostridium* genus and in general, but that it has a vast evolutionary spread across ≥8 bacterial phyla. Motivated by these specific results for *Rho*, the evolutionary behaviour of orthologous families of other prion-like proteins were analysed. Prion-like proteins were defined using compositional criteria and prediction programs for prion domains. Similar evolutionary behaviour to *Rho* was observed for many other bacterial prion-like proteins.

## Methods

### Data

The UniProt [43] set of reference bacterial proteomes (release 2017_12) was downloaded from www.uniprot.org in December 2017, totalling 6469 proteomes.

### Annotation

Various sets of prion-like protein domains were annotated. These were divided into prion prediction (‘PP’) sets, and asparagine/glutamine-rich intrinsically disordered sets (‘NQID’). Prion predictions (PP sets) are the union of annotations made using the programs PAPA and PLAAC with default parameters, except PLAAC was run twice, firstly with budding-yeast background compositions and secondly with the proteome’s own background compositions [17, 18]. For the large-scale analysis of basic statistics, I used a threshold PLAAC log likelihood ratio (LLR) score threshold of ≥20.0, since the lowest value for a known budding yeast prion-forming protein is ~21.0 [25]. Regions biased for glutamine or asparagine residues (i.e., ‘N/Q-rich regions’) were annotated using the fLPS program and a threshold P-value of 1x10^−10^, with background amino-acid frequencies set to be equal (= 0.05) and other parameters set at defaults [44–46]. The WALTZ server for annotating amyloidogenic hexapeptides [47] was also specifically applied to the *Rho* protein orthologs studied for phylogeny as described below. Intrinsically disordered regions were annotated using IUPred and DISOPRED with default parameters [48–50], with a minimum continuous length for the disordered regions of 30 amino acid residues. A 30-residue length cut-off was used since this is a common threshold or boundary value for characterizing intrinsically-disordered regions, or for training algorithms for prediction of intrinsic disorder [51]. Annotated prion-like proteins are provided in the supplementary S1 and S2 Files.

To analyse the evolutionary coverage and cross-phylum spread of prion-like protein status, and their dependence on threshold choice, alternative definitions of prion-like status were used. For the PP prion prediction sets I examined using a low PLAAC LLR threshold of 10.0 (the recently described prion-forming domain in fission yeast protein Ctr4 has a PLAAC LLR score of ~17.0 [14]); I also examined requiring intrinsic disorder for the PPs, as above for N/Q-rich proteins. For N/Q-rich proteins, different lower bias thresholds for N/Q-richness (1x10^−8^ and 1x10^−6^) were also examined.

### Protein clustering and ortholog detection

To remove redundancy from the prion-like protein data sets analysed, they were clustered into families based on analysis of BLASTP [52] output of the protein sequence sets compared to themselves (e-value threshold ≤0.0001 with SEG masking, >0.5 of the sequence length of each protein required to be covered in each sequence alignment), using a script developed by the author [45, 53]. During clustering, sequences are sorted in a list according to decreasing order of their numbers of BLASTP hits, and progressively de-selected as the list is searched. This process yields a list of family *representative sequences* with associated family members.

Every PP or NQID representative protein (from the clustering above) was compared using BLASTP [52] (e-value threshold ≤0.0001, without SEG masking), to all the proteins in each bacterial proteome (whether they are in the PP or NQID sets or not). The bi-directional best hit protein in each proteome was picked for each PP or NQID representative protein to give lists of orthologs. The list of bi-directional best hits was then used to filter the cluster lists and representatives so that they only contain such orthologs.

### Multiple sequence alignment and phylogeny of *Rho*-bot and *Ssb*

Orthologs of the *Clostridium botulinum Rho* protein (‘*Rho*-bot’) were collated by submitting the *Rho* C-terminal domain to the bi-directional best hits method using BLASTP [52] with 357 publicly available proteomes from the *Clostridium* genus, downloaded from the NCBI (www.ncbi.nlm.nih.gov/genome/) in February 2017 (Refseq release 80). Phylogenetic trees were constructed using PhyML, with the aLRT method of branch support [54], based on a multiple sequence alignment made using Clustal Omega [55]. Also, a tree was constructed using the Neighbour-Joining algorithm in the PHYLIP package, with 100 bootstraps [56, 57]. For the further example Ssb (single-strand DNA-binding protein), multiple sequence alignment was performed with Clustal Omega, and phylogenetic analysis with PhyML, as above for *Rho*-bot. Pictures of multiple sequence alignments were drawn using JAlView [58]. The file of *Rho*-bot orthologs is provided in S3 File.

### Calculation of evolutionary coverage

A measure of the evolutionary *coverage* of the prion-like domain was derived. This is defined as the degree to which the prion-like domain arises across its total evolutionary reach within a particular protein family of orthologs. The distance to each ortholog is given by **D** = (1 –**%I**/100.0), where **%I** is the % sequence identity of the match. The *coverage* (**C**) is given by: **C** = (sum of **D** for prion-like orthologs) / (sum of **D** for all orthologs).

A maximum for **D** is set at the highest value observed for any prion-like ortholog of a particular set. This calculation gives an indication of the coverage of the prion-like status within a protein family. The coverage **C** can be considered to be a weighted fraction of the orthologs that have prion-like status. Indeed, for representative proteins with numbers of orthologs >25, **C** is highly correlated with a simple fraction of species having prion-like status (R^2^ ≥ 0.96, C = 0.01); but less so where the number of orthologs is small (< = 25, R^2^ ≤ 0.86). This correlation is likely due to the way in which the set of UniProt reference proteomes sample effectively the diversity in the bacterial evolutionary tree. Also, values of **C** with SEG masking in the BLASTP searches are highly correlated with those where SEG masking is not used (R^2^ ≥0.94, **C**_**seg**_ = 0.94 X **C**_**no-seg**_ for the basic PP set, and C_seg_ = 0.93 X **C**_**no-seg**_ + 0.01 for the basic NQID set).

Also, an alternative calculation of coverage (**C**_**bit**_) was investigated using the bitscore (**B**), with **D** = (**B**–**B**_**min**_), where **B**_**min**_ is the minimum bitscore for a set of orthologs for a representative sequence.

### Gene ontology

Gene Ontology [59] term enrichment was studied for the sets of family representative sequences using a normal approximation to binomial probability, with a Bonferroni correction for multiple hypothesis testing (P-value threshold = 0.000017). The background population for testing was a set of representative sequences derived by clustering a 5% sample of the total set of bacterial proteins from the reference proteomes (see S4 File for further details).

## Results & discussion

An analysis of the phylogenetic penetrance of the *Clostridium* transcription termination factor *Rho* (abbreviated ‘*Rho*-bot’) prion-like domain is described, *i*.*e*., how evolutionarily broad is the distribution of the domain, and is its conservation deep or sporadic? Motivated by the results of this analysis, I then investigated the distribution of prion-like proteins amongst different species and phyla of bacteria. I surveyed two aspects of the evolutionary behaviour of these domains: firstly, the degree of spread of particular prion-like domain families across multiple phyla; secondly, how much the prion-like domain is conserved across organisms within its evolutionary range, *i*.*e*., its evolutionary ‘coverage’.

### Evolutionary analysis of transcription activator *Rho*

**Fig 1:**
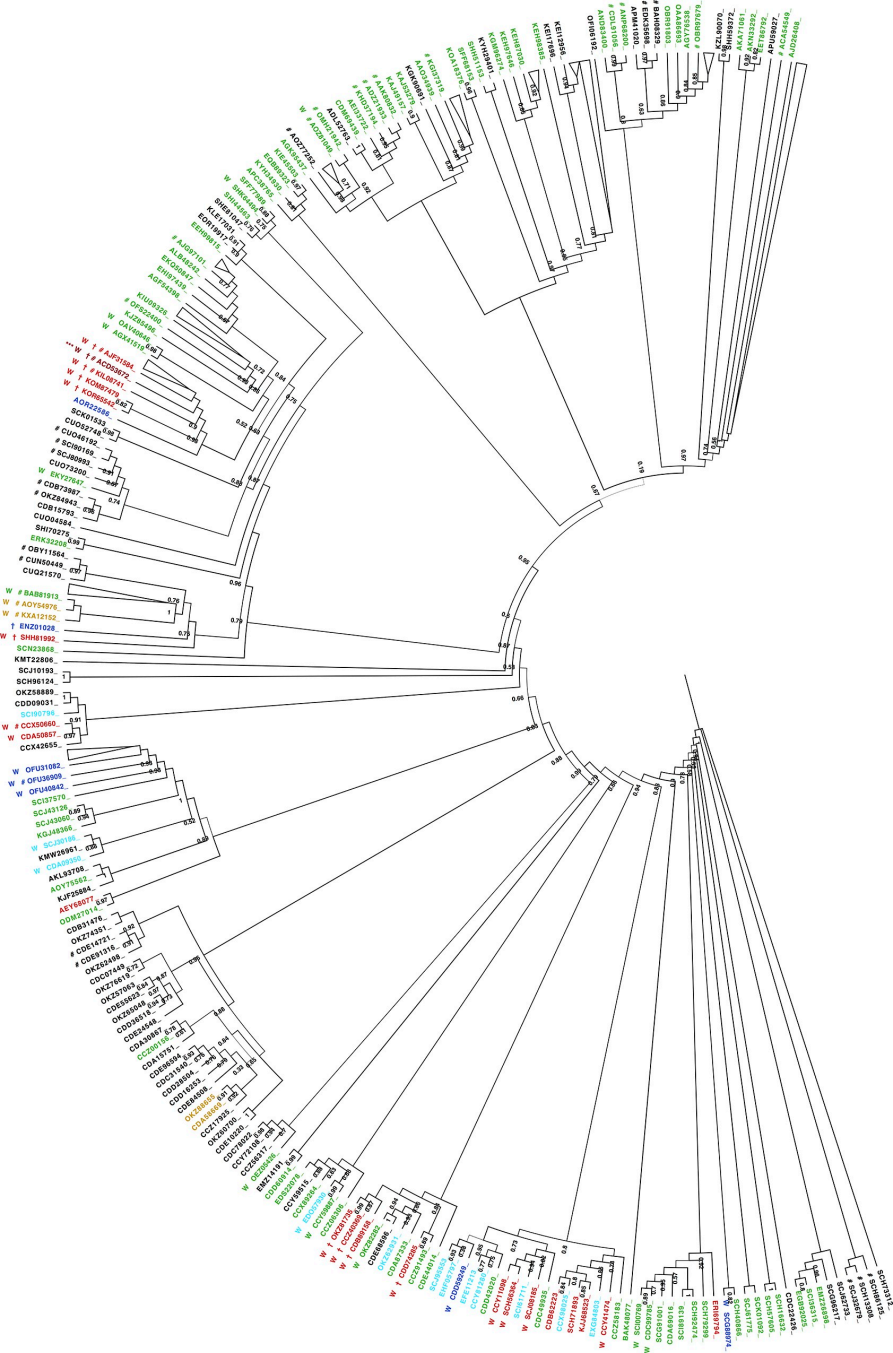
A phylogenetic tree of transcription termination factor *Rho* for the *Clostridium* genus. This tree was constructed using PhyML [54], based on a multiple sequence alignment of 357 orthologs made using Clustal Omega [55], as described in *Methods*. The nodes are labelled with their PhyML approximate likelihood ratio test support values, if they are >0.5. Sequences that are identical to their most immediate neighbours in the tree are labelled with ‘#’, with large clusters of identical sequences collapsed to a wedge symbol. The tree was drawn using FigTree [61]. PLDs were identified using a PLAAC threshold for LLR score ≥20 [25]. These have their NCBI identifiers coloured red. Other sequences that have LLR score ≥10.0 are coloured light blue, and any further cases with LLR ≥0.0 are coloured dark blue. Intrinsically disordered proteins that are also N/Q-rich according to the fLPS program (with default parameters), are coloured orange [44]. Other proteins with an N-terminal intrinsically disordered domain ≥30 residues are coloured green. Proteins that also have PAPA scores ≥0.05 are labelled with a ‘†’ symbol [18]. Proteins that are also predicted by the Waltz prediction server to have amyloidogenic peptides in their intrinsically disordered regions are labelled with a ‘W’ [47]. The *C*. *botulinum* strain *Rho* studied for prion formation marked with ‘***’. The set of five prion predictions around this protein that are coloured red form the *C*. *botulinum* cluster. The same tree is produced, with <10 variant taxa using the neighbour-joining algorithm of the PHYLIP package (bootstrapped 100 times) [56, 57].

A complete phylogeny of *Rho* from available *Clostridium* genus proteomes was constructed. This was labelled using a variety of programs for annotating predicted prions, amyloidogenicity, intrinsic disorder and compositional bias (Fig 1). This labelled phylogeny demonstrates that the prion-like domain (PLD) appears sporadically in this genus, with a lack of conservation of prion-like character suggesting that this character *per se* may not be necessary for essential *Rho* protein function. The *Rho*-bot PLD—which is a very simple, homopeptide-rich sequence—appears to have arisen recently in an early ancestor of the *C*. *botulinum* species and elsewhere sporadically in the genus, *i*.*e*., there is no deep conservation of prion-like character, and there is often an intrinsically disordered region with different composition in the same place in the protein sequence (Fig 1). The N-terminus of the protein is well conserved (S5 File), indicating that the lack of PLDs is not due to genome mis-annotation. Such sporadicity may be linked to the potent nature of prion propagation, meaning that prion-forming proteins may be tolerated or remain useful for short evolutionary timespans of several millions of years, since their propagation may arise so rarely in wild bacterial populations, but thereafter the prion domains could be subsequently purged from the proteins if they lose their utility or become detrimental to fitness [60]. Alternatively, the intrinsically disordered region may have a different function that allows more variation over a wide amino-acid compositional range, including a ‘prion-like’ composition.

**Fig 2:**
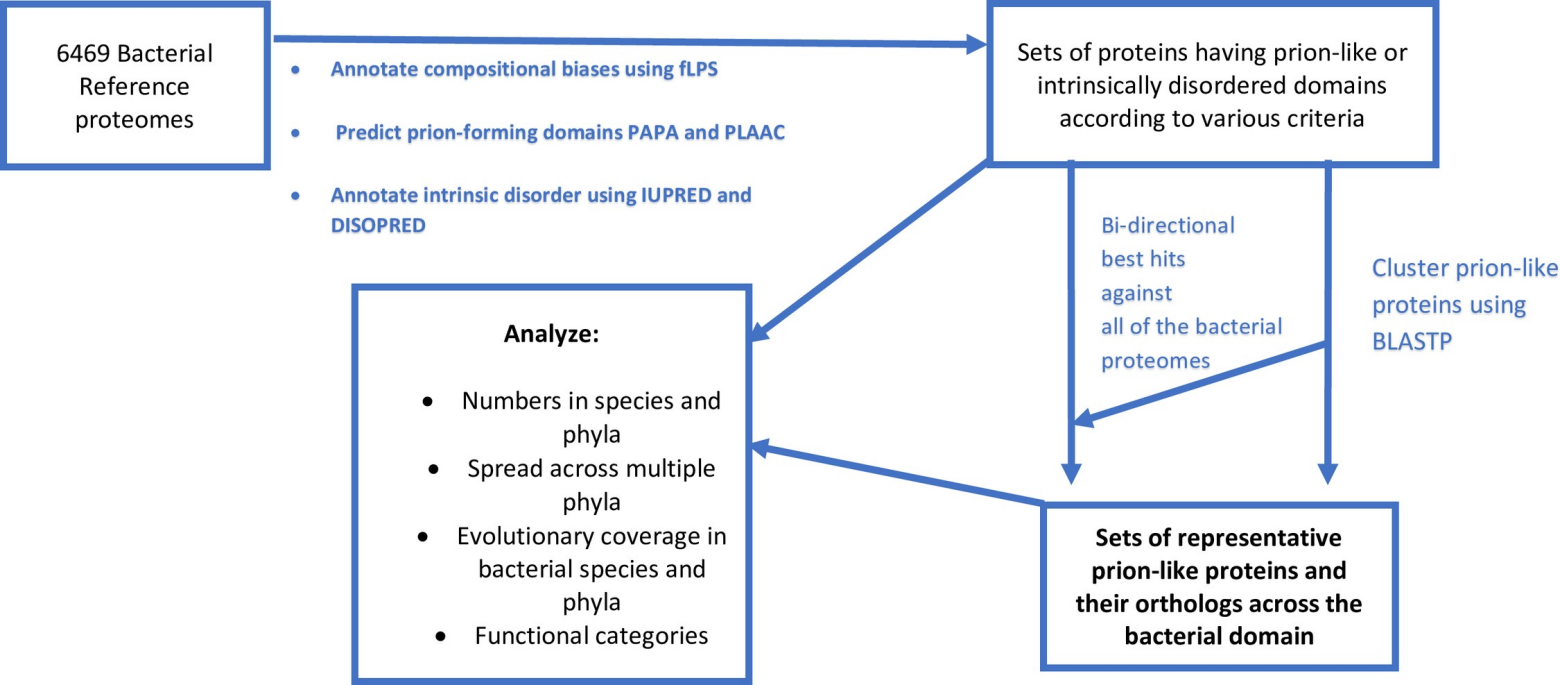
A schematic of the analysis performed on bacterial proteomes for the evolutionary penetrance of prion-like proteins.

Is such evolutionary sporadicity coupled with wide evolutionary spread a general characteristic of prion-like proteins in bacteria, or are there also more deeply conserved cases? To address this question, I performed an analysis of the evolutionary reach and sporadicity of *Rho* and a large list of thousands of prion-like proteins across the whole of the bacterial domain, as summarised in Fig 2. Both N/Q-rich disordered (‘NQID’) and prion prediction (‘PP’) sets of proteins were analysed.

### Summary of the distribution of prion-like proteins in bacteria

**Fig 3:**
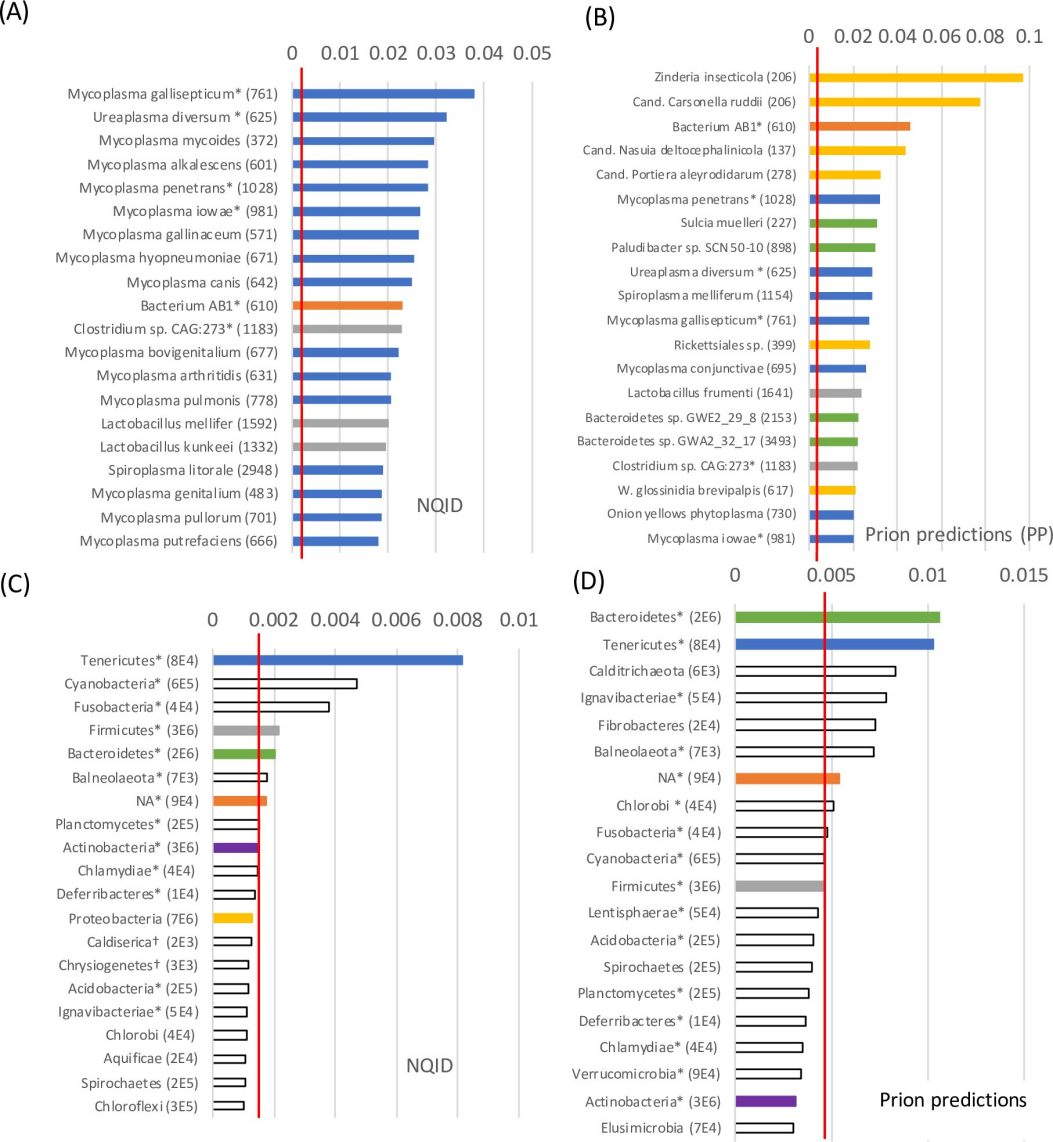
The phyla and species with the highest fractions of prion-like proteins. In (A) and (C), the twenty species and phyla with the highest fractions of prion-like proteins (NQID set) are shown. In (B) and (D), similarly are shown the corresponding lists for the PP set. Parts (C) and (D) also act as color keys for the phyla of the species listed in parts (A) and (B). ‘NA’ means un-classified in any phylum. The total numbers of prion-like proteins of either set are given in brackets after the species or phylum name. Species/phyla that are in both top twenty lists are asterisked. The average fraction of prion-like proteins in either data set is indicated by the red line in each bar chart.

Prion-like proteins are a small fraction of bacterial proteomes (Table 1). The largest fractions of NQID proteins are in the *Mycoplasma* genus (phylum *Tenericutes*), which also has many prion predictions (Fig 3). *Mycoplasma* is an important pathogenic genus that has extensive antibiotic resistance. Also, there are specific species from the phyla *Bacteroidetes*, *Firmicutes* and *Proteobacteria* with high fractions of prion predictions (>2%) in their proteomes, such as *Zinderia insecticola*, a symbiont of spittlebugs with a tiny genome [62] (Fig 3B). The *Bacteroidetes* and *Tenericutes* phyla have the highest fractions of prion predictions (Fig 3D).

**Table 1:** Summary of data sets.

*Number of representative proteomes* *= 6469*							*Number of proteins in representative proteomes = 1*.*86x10*^*7*^
OVERALL STATISTICS							
Set	Total number of prion-like proteins				Ratio per proteome	Ratio per protein	Number of families †
**NQID:** Proteins with N/Q-rich regions (N/Q compositional biased, intrinsic disorder)	27472	N-rich*	Q-rich*	N+Q-rich*	4.3	0.0015	438
		11296	16586	251			
**PP:** Prion predictions by either PAPA or PLAAC	70942	PLAAC**		PAPA**	11.0	0.0038	520
		20699		53213			

† The number of families derived as described in methods, that have ≥5 members.

* The subset of the NQID set that are N-rich, Q-rich or (N+Q)-rich (according to the same thresholds as for the overall NQID set.

** The subset of the PP set that are predicted by PLAAC, or by PAPA (according to the same thresholds as for the overall PP set).

The NQID set is comprised mostly of Q-rich regions (60%), with only a small fraction biased for glutamine and asparagine in combination (1%) (Table 1). The PP set is mostly comprised of PAPA predictions (75%) (Table 1). The sequences for these NQID and PP sets are in S1 and S2 Files.

To remove redundancy in the counting of proteins in the basic NQID and PP sets (Table 1), they were clustered into >400 families of at least five members. Of course, such families might actually be larger and spread more extensively over multiple phyla (and thus the number of families in Table 1 smaller); here, however, the goal is to use these family clusters to reduce redundancy in the statistics of these proteins. These values also provide upper-bound estimates for the total number of families of prion-like proteins in the bacterial domain, for the specific criteria for prion-like status used.

The major Gene Ontology functional enrichments for bacterial prion predictions are ‘receptor activity’, ‘self-proteolysis’ and ‘outer membrane’ (S4 File) [59]. ‘Single-stranded DNA binding’ stands out as common to both the NQID and Prion Prediction lists. The proteins here that undergo self-proteolysis have an RHS repeat-containing core domain, which functions as a self-cleaving protease; it is interesting that this process might interface with prion-like aggregation in some way. An enzymatic prion of vacuolar protease B was described in budding yeast, wherein the active state of protease B causes its own propagation [63]. Formation of prion-like aggregates by ‘outer membrane’ or ‘receptor’ proteins may be used functionally to propagate signalling cascades, such as is observed for human MAVS protein [64]. Previous analysis of GO terms was performed on data sets of prion predictions without prior clustering to remove redundancy [40], so the lists are not directly comparable (Table B in S4 File).

### Evolutionary coverage of prion-like proteins

**Fig 4:**
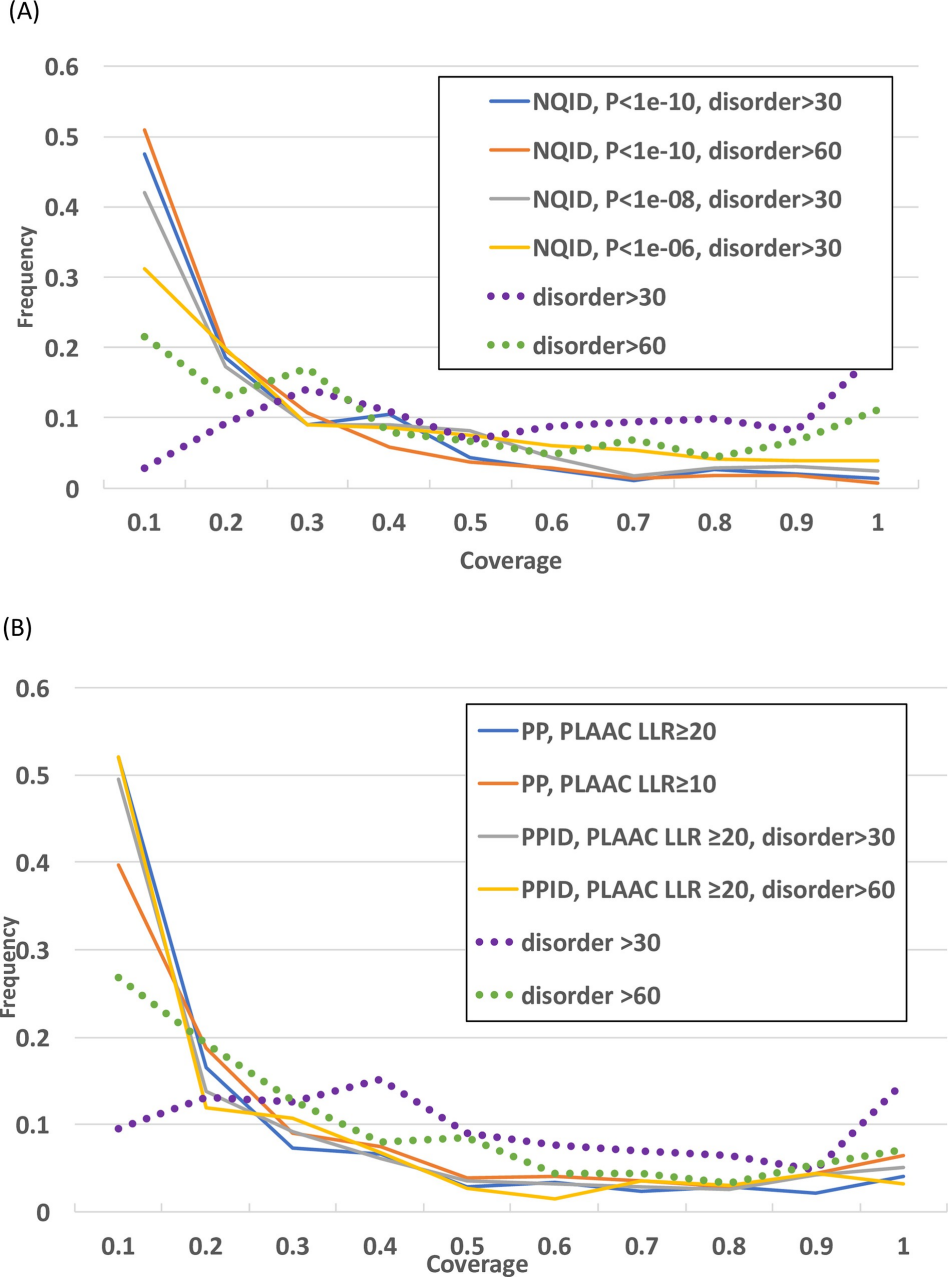
The coverage of prion-like proteins within their evolutionary range. Only families of proteins with ≥5 prion-like proteins are considered. The acronym NQID stands for N/Q-rich intrinsically disordered. The distributions for disorder only are just from annotations with the program IUPRED. (A) The distribution of coverage (*C*) for the NQID sets with various parameters as listed: P-value for bias annotation by the fLPS program; length of intrinsic disorder as measured by either the IUPRED or DISOPRED programs. (B) The distribution of coverage (*C*) for the prion prediction sets with various parameters as listed: log likelihood ratio (LLR) score for PLAAC prion-forming region annotation; length of intrinsic disorder as measured by either the IUPRED or DISOPRED programs.

A measure of evolutionary coverage **C**, a weighted fraction of the orthologs that have prion-like status within the evolutionary range of this status, was calculated to check whether apparent sporadicity of prion-like domains is a general behaviour in bacteria. The distribution of **C** was derived for various definitions of NQID and PP sets, and compared to corresponding distributions of **C** for intrinsic disorder (Fig 4). Regardless of definition, prion-like status tends to have low coverage across the evolutionary range of specific proteins. For example, for the basic NQID and PP sets, 48% and 52% have **C** ≤0.1. For a speculative lower threshold of PLAAC prion prediction LLR score = 10.0, this decreases to 40% for the PP set. Mean **C** values for any of the NQID or PP sets are highly significantly less than corresponding **C** values for intrinsic disorder (P<<0.000001 for unpaired t-tests). Similarly, PP sets generally have less coverage than NQID sets (highest P-value for any comparison = 0.045, with the exception of the NQID set with P-value threshold 1e-06). Similar trends for comparing the basic NQID / PP sets with their corresponding disorder are observed if coverage is calculated using BLASTP bit scores (**C**_**bit**_ distributions, S6 File).

### Phylogenetic spread of prion-like proteins across multiple phyla

**Fig 5:**
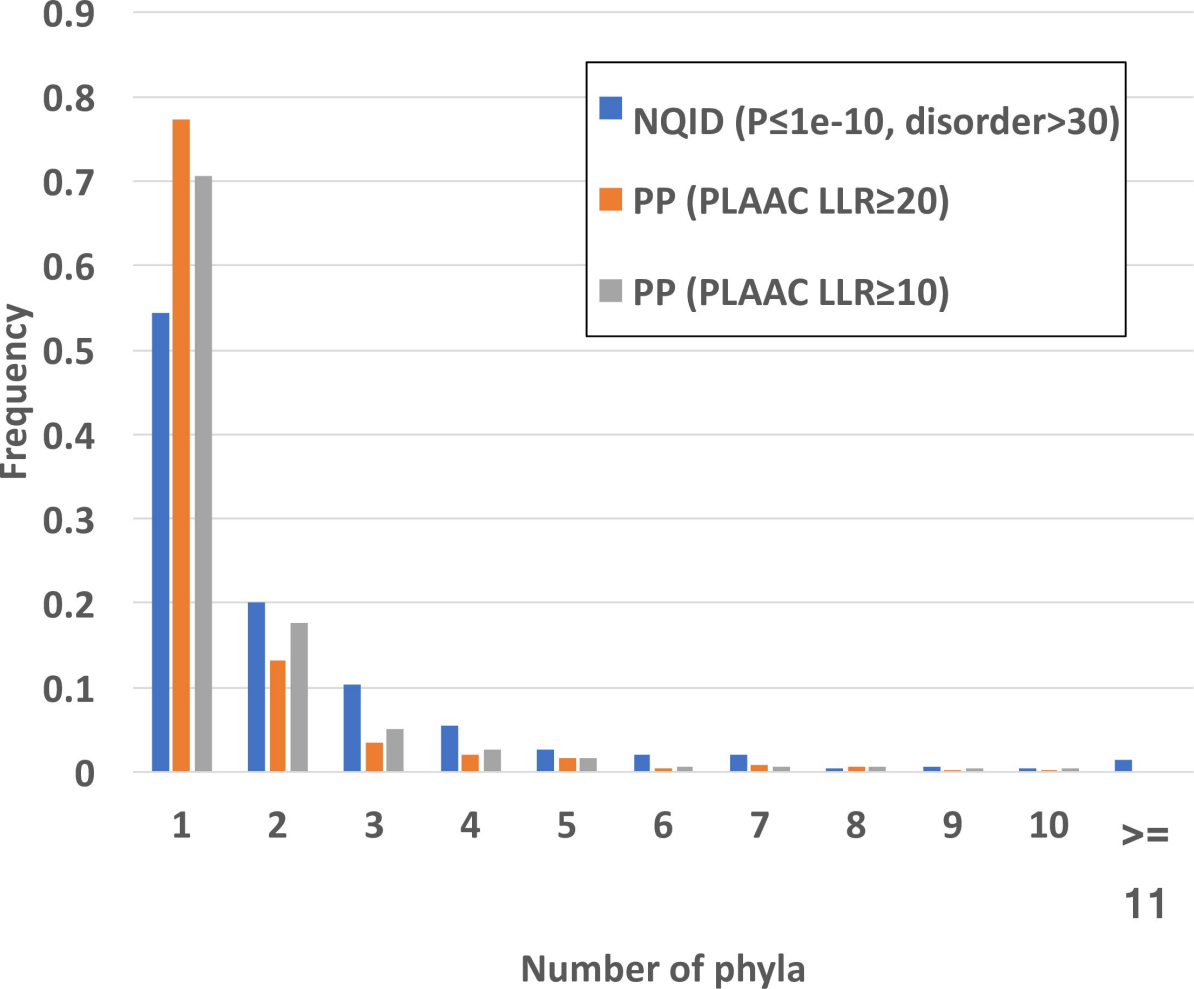
Cross-phylum distribution of prion-like protein families. The cross-phylum distribution of the prion-like protein family representative sequences is shown for prion prediction and NQID sets (as detailed in *Methods*).

I examined the number of phyla in which orthologous prion-like protein families are observed (Fig 5). A large fraction of them occur in multiple phyla (46% for the basic NQID set examined, and >23% for the PP sets). These percentages can be considered lower bounds for cross-phylum spread, since the families are likely under-clustered. There is little difference in the cross-phylum spread of the PP sets with PLAAC LLR thresholds set either at 10.0 or at 20.0 (23–29%); also, using different NQID bias thresholds results in no difference in the phylum distribution, indicating that thresholds are not an issue for spread across multiple phyla.

In Table 2 are listed the top three families of the basic NQID and PP sets ranked in terms of number of phyla in which they occur. Transcription termination factor *Rho* is highly ranked for either set (#3 for PP and #9 for NQID). The most widespread NQID protein is chromosome partition protein *Smc*, which functions in chromosome condensation and partitioning. Translation initiation factor IF-2 has a predicted prion-forming domain with higher coverage than *Rho* across approximately a tenth of the protein’s evolutionary range. Of course, this possible prion-forming domain falls in with a trend for known prion-forming domains involved in control of translation and transcription, *e*.*g*., Sup35p or Sfp1p in *Saccharomyces cerevisiae* [2, 65].

**Table 2:** Lists of the top-three families that occur across the greatest number of phyla, for both NQID and prion prediction sets of proteins.

Ranking	Rep. example	Description	# of Phyla	# of prion-like orthologs	Coverage value (C)*
**NQID basic set**					
**#1**	A0A0R2CDR3	chromosome partition protein Smc	**17**	970	0.210
**#2**	A3WQSF6	Chromosome segregation ATPase Sms	**12**	192	0.178
**#3**	Q6AG72	Nuclease SbcCD	**12**	313	0.063
**#9**	N2BSW8	Transcription termination factor *Rho*	**9**	345	0.069
**Prion predictions (PP) basic set**					
**#1**	A0A1F3NRB5	Tetratricopeptide repeat protein	**10**	90	0.169
**#2**	I4D9F1	Translation initiation factor IF-2	**9**	548	0.103
**#3**	N2BSW8	Transcription termination factor *Rho*	**8**	304	0.062

* The coverage for NQID or PP as appropriate.

Generally, there are very few prion-like families that have high coverage (**C** ≥0.5) and that occur in large numbers of phyla or species (Table 3). For example, there are 61 PP families that demonstrate C values greater than or equal to that observed for *Rho* protein, and that occur in multiple phyla. The prion-like families analysed in this table are listed in S7 and S8 Files. One notable example is *Ssb*, single-stranded DNA-binding protein, which has a prion-like domain at its C-terminus in 802 orthologous members of its family, with **C** = 0.129. The protein *Ssb* binds to single-stranded regions of DNA and functions in replication, recombination and DNA repair. This particular prion-like domain family has the highest number of orthologs (totalling 68) that are predicted as prions by both the PLAAC and PAPA algorithms. They spread across three phyla. This analysis is presented in the supplementary S9 File.

**Table 3:** Numbers of prion-like protein families (basic PP set) for various criteria.

Coverage (C) →	≥0.06 *	≥0.50	≥0.75
**Number of phyla**			
**≥2**	61	11	1
**≥3**	32	5	0
**≥5**	18	2	0
**Number of species**			
**≥25**	126	31	10
**≥50**	77	25	9
**≥100**	35	8	3

* Value of C for *Rho* transcription termination factor.

### Concluding remarks

Regardless of the thresholds for prion or N/Q-rich domain definition examined, we see a general pattern of apparent sporadic conservation for prion-like domains across bacteria, particularly in comparison to corresponding intrinsically disordered regions in the same proteins. This is often coupled to a wide distribution across multiple phyla. These results motivate several hypotheses. Prion propagation may arise rarely enough in wild bacterial populations that prion-like domains are maintained in specific clades of organisms for millions of years [60]. They may occasionally be beneficial, but then may also occasionally become detrimental to fitness, and so be subsequently purged. Purging may involve accumulation of a small number of sufficient mutations to avoid detrimental frequent aggregate formation [66], followed by mutational drifts in intrinsically disordered regions of the sort evidenced in analyses of prion-like domains in fungi [25]. Also, in some cases, such domains, either in bacteria or eukaryotes, may be shifting to undiscovered alternative compositions for prion-like domains; however, this phenomenon has yet to be detected for any known prion domain.

The results here provide a test list for further experimental investigation of possible prion-like domains in bacteria, which form a potentially significant part of protein ‘dark matter’, the un- or under-appreciated parts of proteins that remain to be characterized in the protein universe [45]. For example, there are 35 prion-like domain families that occur in ≥100 species and have at least the evolutionary coverage of the *Rho* prion-like domain family. The methodology for calculating evolutionary coverage can also be applied to the evolutionary behaviour of other types of protein domain/region.

## Supporting information

S1 File FASTA-format text file containing the annotated basic NQID protein set in bacteria. A header is added to the top of the file that explains the format of the labels and names for each sequence.
(FASTA)

S2 File FASTA-format text file containing the annotated basic PP (prion prediction) protein set in bacteria. A header is added to the top of the file that explains the format of the labels and names for each sequence.
(FASTA)

S3 File FASTA-format file of the orthologs of *Rho*-bot. The species are listed on the ‘>‘ name lines.
(TXT)

S4 File Analysis of Gene Ontology (GO) categories for prion-like proteins in bacteria. (DOCX)

S5 File Multiple sequence alignment for the N-terminal part of *Rho*-bot. (DOCX)

S6 File Coverage of prion-like proteins within their evolutionary range, using the bitscore to calculate coverage (C_bit_). (PDF)

S7 File Table of C coverage values for clusters that occur in more than phylum, and have at least C = 0.06 (the value for Rho-bot). The file format is explained in the header in the file.
(TXT)

S8 File Table of C coverage values for clusters that occur in ≥25 species, and have at least C = 0.06 (the value for Rho-bot). The file format is explained in the header in the file.
(TXT)

S9 File Example of predicted prion protein (Ssb, single-stranded DNA–binding protein) that is conserved across multiple phyla, with a prion-like domain identified by both prion annotation programs employed. (PPTX)
