# Supplementary material for: Evolutionary behaviour of bacterial prion-like proteins
Source: PLoS One. 2019 Mar 5;14(3):e0213030. doi: 10.1371/journal.pone.0213030 (PMC6400439; doi:10.1371/journal.pone.0213030)
Supplement: S4 File — (DOCX) [file pone.0213030.s004.docx]

**S4 File**

Gene Ontology category enrichments were analysed for all three ontologies (biological process, component and molecular function). A Bonferroni correction for the significance threshold was used. A normal approximation to binomial probability was used to calculate P-values (z-score threshold = 4.14, P-value = 0.000017), because of the large size of the background populations. Here analyzed were sets of bacterial prion-like protein cluster representatives for clusters of size ≥5 derived as described in *Methods* in the main text (Table 1). In part (A), the top ten categories for the basic set of NQIDs are listed, and in part (B) for the basic set of PPs. Categories are in bold text if they are enriched for both sets.

**Table 1: Enrichments for complete sets of prion-like proteins**


| **Representative sequences in the NQID set (total = 438)** | | |
| --- | --- | --- |
| **GO term** | **#** | **Z-score** |
| GO:0003774 motor activity | 5 | 13.2 |
| GO:0007062 unfolded protein binding | 8 | 12.6 |
| GO:0005618 cell wall | 6 | 11.9 |
| **GO:0003697 single-stranded DNA binding** | **4** | **10.5** |
| GO:0030983 mismatched DNA binding | 3 | 9.9 |
| GO:0006298 mismatch repair | 3 | 8.8 |
| GO:0004527 exonuclease activity | 7 | 8.6 |
| GO:0004518 nuclease activity | 9 | 7.8 |
| GO:0051301 cell division | 9 | 7.3 |
| GO:0004521 endoribonuclease activity | 3 | 7.2 |

(B)

| Representative sequences in the prion prediction data set (total = 520) | | |
| --- | --- | --- |
| GO term | # | Z-score |
| GO:0097264 self-proteolysis | 12 | 21.1 |
| GO:0004872 receptor activity | 18 | 16.5 |
| GO:0019867 outer membrane | 13 | 11.7 |
| GO:0004040 amidase activity | 6 | 11.6 |
| GO:0009253 Peptidoglycan catabolic process | 9 | 10.4 |
| GO:0000272 polysaccharide catabolic process | 8 | 10.1 |
| **GO:0003697 single-stranded DNA binding** | **4** | **9.5** |
| GO:0003796 lysozyme activity | 4 | 9.3 |
| GO:0009279 cell outer membrane | 12 | 9.1 |
| GO:0016998 cell wall macromolecule catabolic process | 4 | 9.0 |
