# Supplementary material for: Evolutionary behaviour of bacterial prion-like proteins
Source: PLoS One. 2019 Mar 5;14(3):e0213030. doi: 10.1371/journal.pone.0213030 (PMC6400439; doi:10.1371/journal.pone.0213030)
Supplement: S5 File — (DOCX) [file pone.0213030.s005.docx]

**S5 File**

Absence of a PLD is not due to truncation of mis-annotated proteins at the N terminus, since N terminal sequences align well across almost all of the *Clostridium* species. There is dark blue shading for residues that agree with the consensus residue (most common residue) for each column, and light blue for residues that have a positive BLOSUM62 score relative to consensus *(13)* (drawn with Jalview *(14)*). The identifiers of proteins are prefixed with a ‘P’ if they contain a prion-like domain, and with ‘N’ if not.
