# Supplementary material for: Evolutionary behaviour of bacterial prion-like proteins
Source: PLoS One. 2019 Mar 5;14(3):e0213030. doi: 10.1371/journal.pone.0213030 (PMC6400439; doi:10.1371/journal.pone.0213030)
Supplement: S6 File — (PDF) [file pone.0213030.s006.PDF]

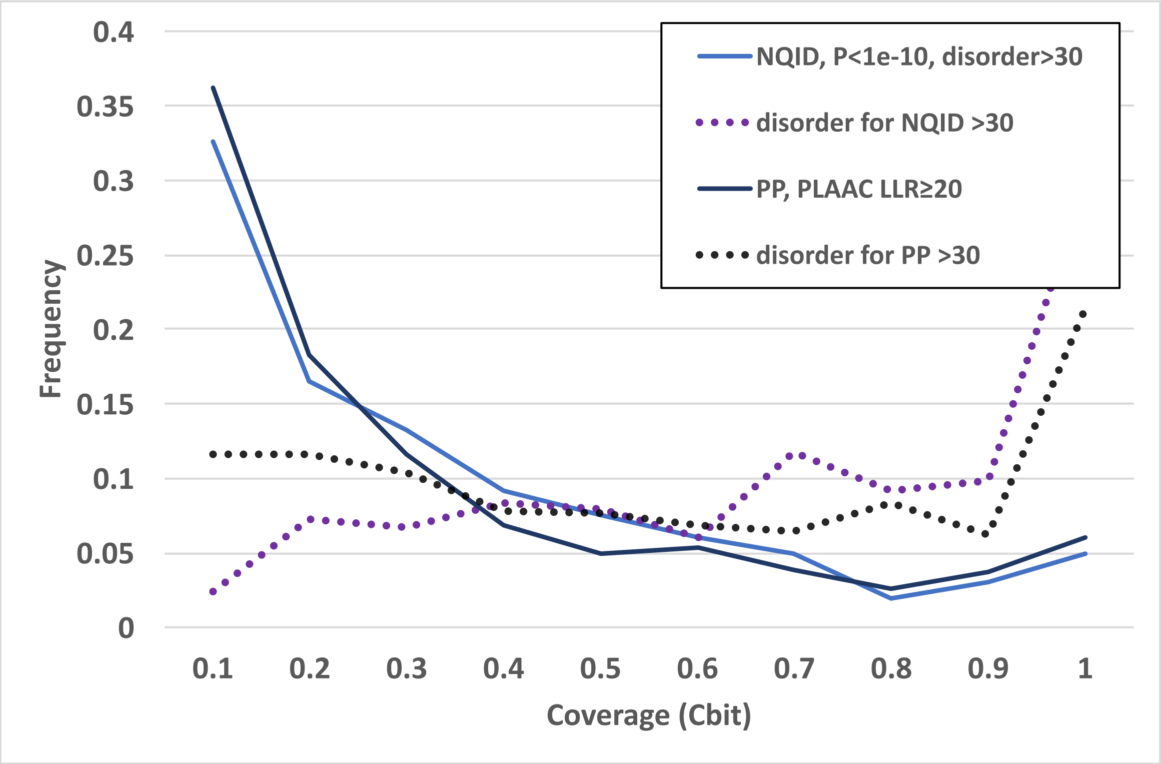

S6 File The distribution of coverage (*C*) for the basic NQID and PP sets with various parameters as listed (P-value for fLPS bias annotation; length of intrinsic disorder as measured by IUPRED or DISOPRED). The corresponding distributions for disorder (from IUPRED annotations) are also shown. Only families of proteins with ≥5 prion-like proteins are considered. Set acronyms are as described in *Methods* main text.
