# Supplementary material for: Evolutionary behaviour of bacterial prion-like proteins
Source: PLoS One. 2019 Mar 5;14(3):e0213030. doi: 10.1371/journal.pone.0213030 (PMC6400439; doi:10.1371/journal.pone.0213030)
Supplement: S9 File — (PPTX) [file pone.0213030.s009.pptx]

## Slide 1
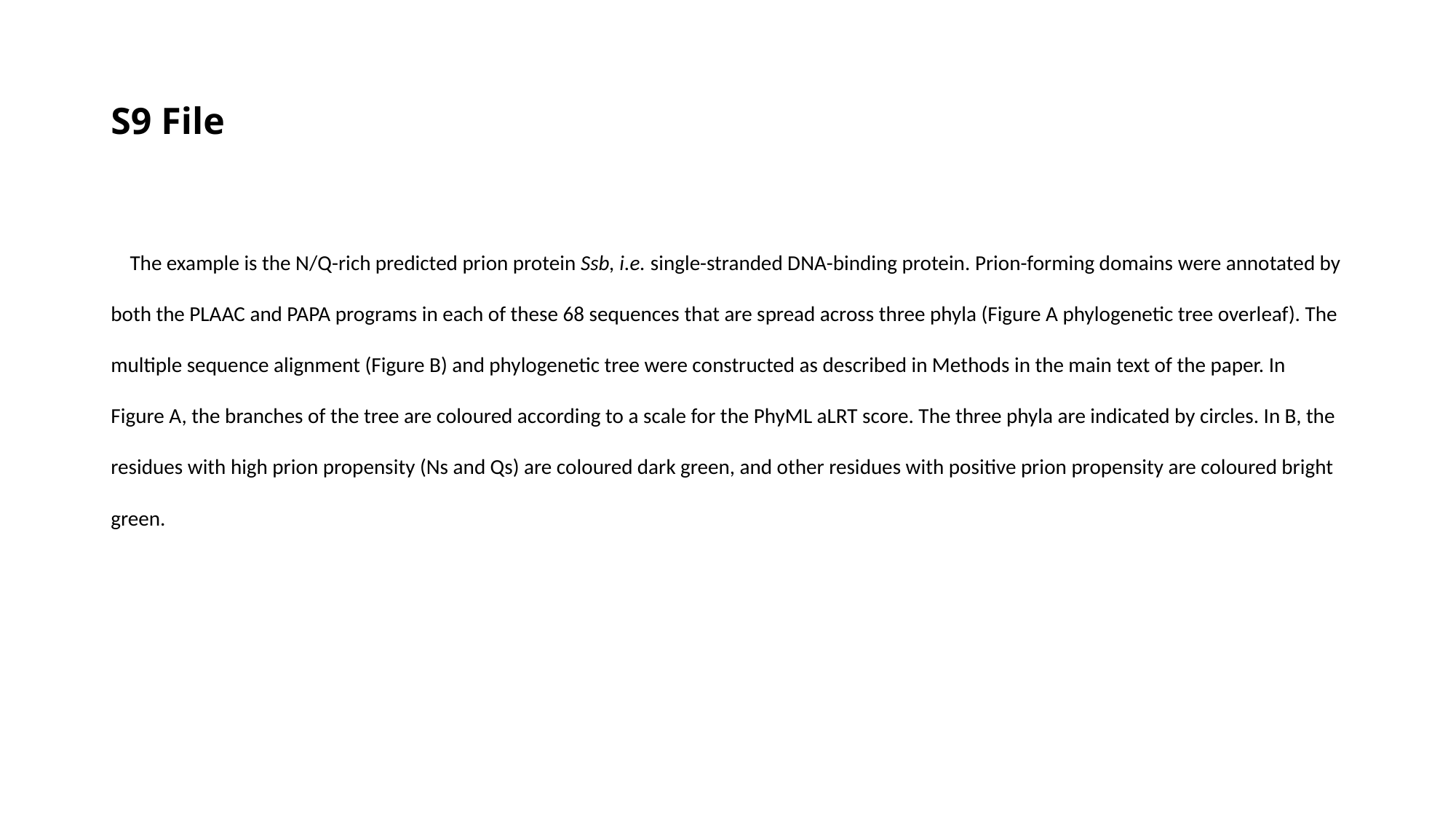

# S9 File
 The example is the N/Q-rich predicted prion protein Ssb, i.e. single-stranded DNA-binding protein. Prion-forming domains were annotated by both the PLAAC and PAPA programs in each of these 68 sequences that are spread across three phyla (Figure A phylogenetic tree overleaf). The multiple sequence alignment (Figure B) and phylogenetic tree were constructed as described in Methods in the main text of the paper. In Figure A, the branches of the tree are coloured according to a scale for the PhyML aLRT score. The three phyla are indicated by circles. In B, the residues with high prion propensity (Ns and Qs) are coloured dark green, and other residues with positive prion propensity are coloured bright green.

## Slide 2
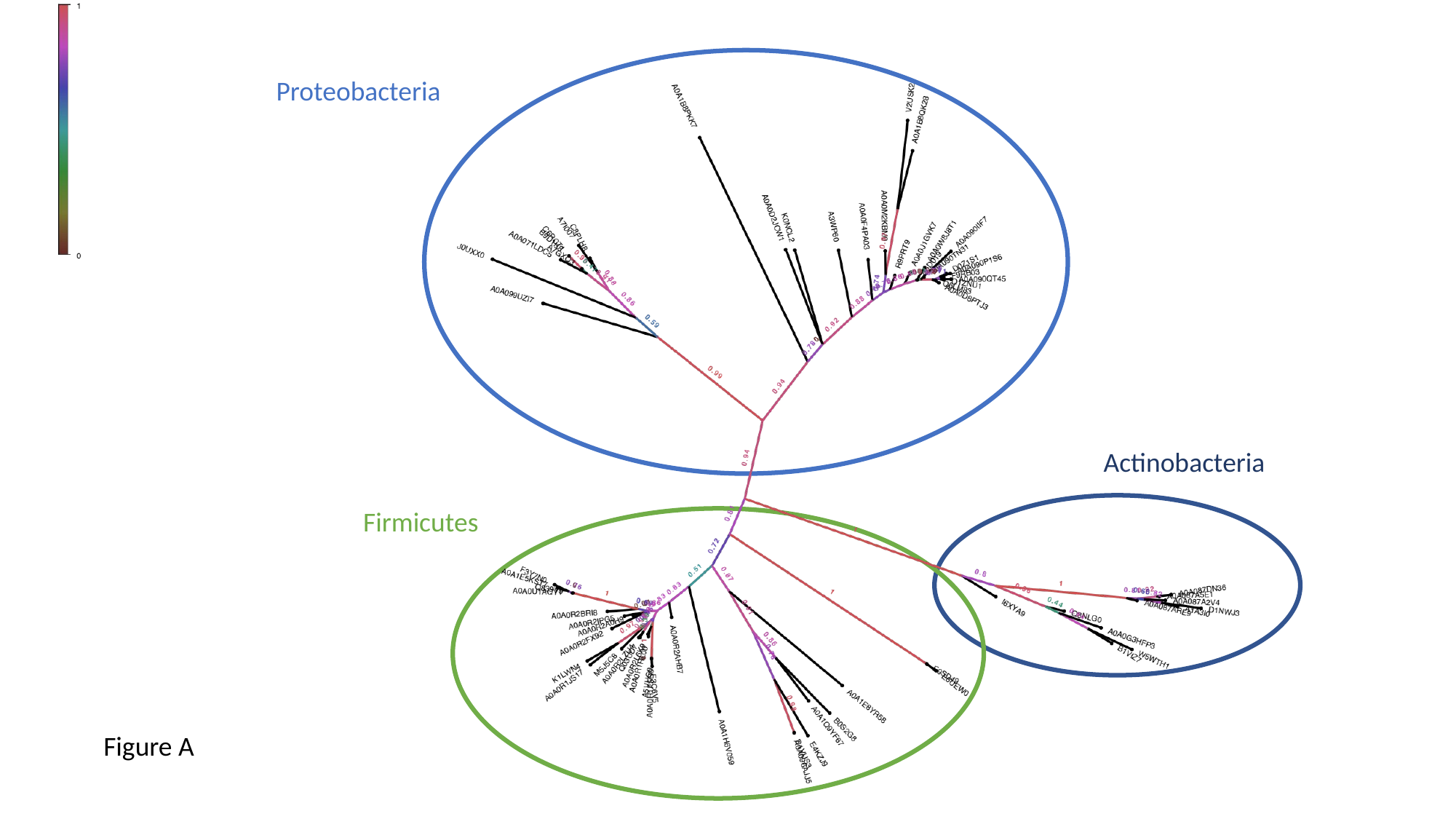

Proteobacteria
#
Actinobacteria
Firmicutes
Figure A

## Slide 3
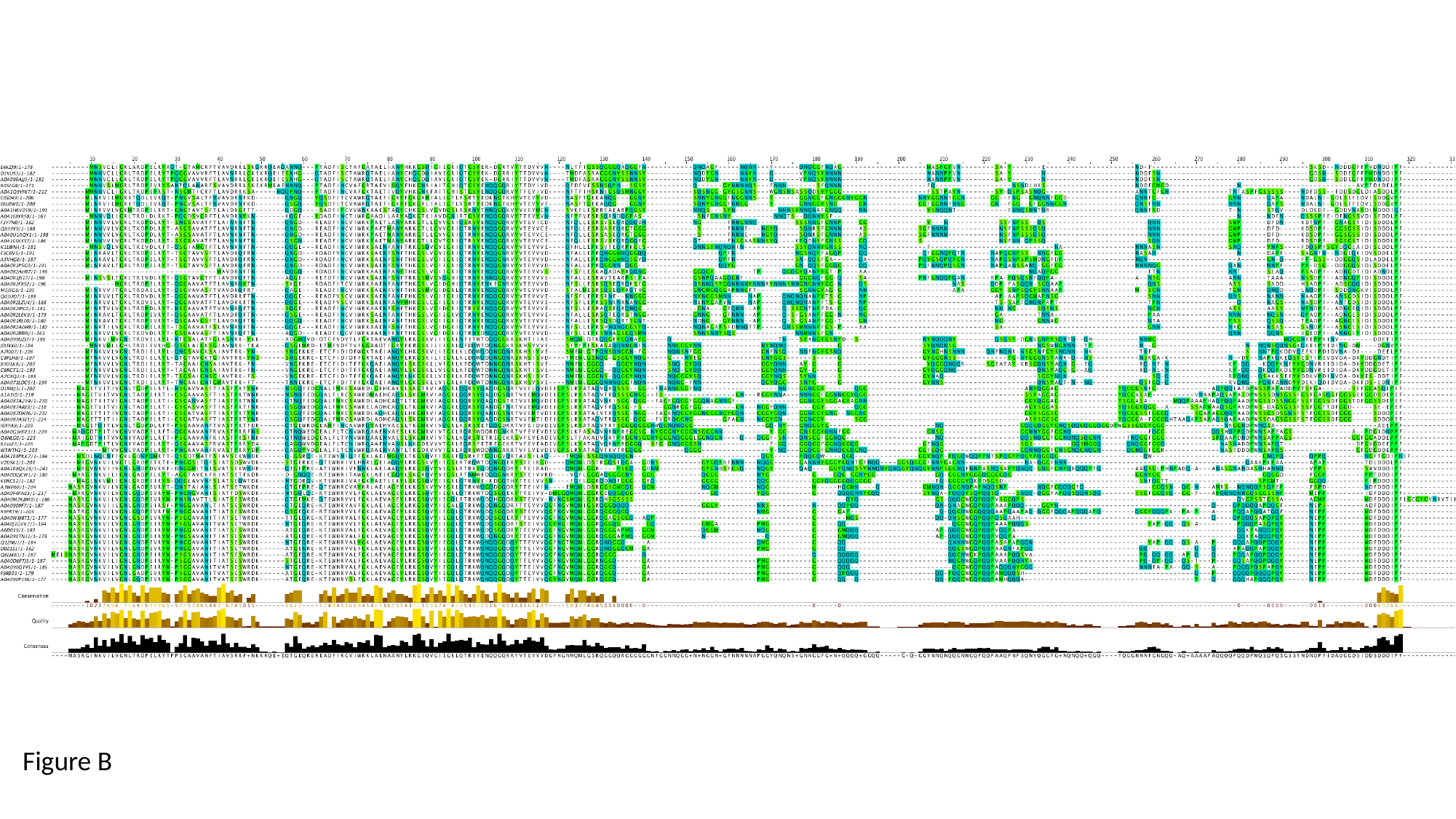

#
Figure B
